# Transcriptomic Signatures of Mitochondrial Dysfunction in Autism: Integrated mRNA and microRNA Profiling

**DOI:** 10.3390/genes16091065

**Published:** 2025-09-10

**Authors:** Richard E. Frye, Zoe Hill, Shannon Rose, Sandra McCullough, Patricia A. Porter-Gill, Pritmohinder S. Gill

**Affiliations:** 1 Autism Discovery and Treatment Foundation, Phoenix, AZ 85050, USA; zoe@autismtx.org; 2 Department of Health Promotion and Disease Prevention, College of Nursing, The University of Tennessee Health Science Center, Memphis, TN 38163, USA; srose37@uthsc.edu; 3 Arkansas Children’s Research Institute, Little Rock, AR 72202, USA; mcculloughsandras@uams.edu (S.M.); pportergill@gmail.com (P.A.P.-G.); satnampsgill@gmail.com (P.S.G.); 4 Department of Pediatrics, University of Arkansas for Medical Sciences, Little Rock, AR 72202, USA

**Keywords:** autism spectrum disorder, cell growth, CamKinase II, microRNA, mitochondria, mTOR

## Abstract

Background: Prior work established that about a third of ASD-derived LCLs show excessive mitochondrial respiration and stress vulnerability—features divergent from both controls and classical mitochondrial disease. This study explores how mRNA and microRNA (miRNA) expression profiles distinguish subtypes of autism spectrum disorder (ASD) defined by mitochondrial function. Methods: Lymphoblastoid cell lines (LCLs) from boys with ASD were classified into two groups: those with abnormal (AD-A) and normal (AD-N) mitochondrial function. RNA-seq compared mRNA and miRNA expression differences. Results: 24 mRNA differentially expressed genes (DEGs) (14 downregulated, 10 upregulated in AD-N vs. AD-A) were identified, implicating processes such as mRNA processing, immune response, cancer biology, and crucially, mitochondrial and nuclear activities. Notably, genes such as DEPTOR (an mTOR modulator) were upregulated in AD-A, highlighting dysregulation in the mTOR pathway—a central regulator of cellular metabolism, protein synthesis, autophagy, and mitochondrial function. miRNA analysis revealed 18 differentially expressed miRNAs (DEMs) upregulated and one downregulated in AD-N compared to AD-A. Several miRNAs (including hsa-miR-1273h-3p, hsa-miR-197-3p, and hsa-miR-199a-5p) targeted both the differentially expressed genes and pathways previously linked to ASD, such as mTOR, Calmodulin Kinase II, and mitochondrial regulation. Enrichment analyses indicated involvement regulation of cell growth and division, gene expression, immune regulation and cellular stress as well as mTOR signaling. Conclusions: These molecular signatures support the idea that mitochondrial dysfunction in ASD is tied to specific disruptions in the mTOR and PI3K/AKT signaling axes, influencing cell growth, autophagy, oxidative stress handling, and neuronal metabolism. The findings highlight a miRNA-mRNA regulatory network that may underpin mitochondrial dysfunction and ASD heterogeneity, suggesting avenues for subtype-specific biomarkers and targeted therapies that address energy metabolism and cellular stress in ASD.

## 1. Introduction

Autism Spectrum Disorder (ASD) is a complex neurodevelopmental disorder characterized by deficits in social-communication and the presence of restricted, repetitive and inflexible behaviors and interests [1]. ASD affects one in 31 children in the United States (US) and with boys being effected four times more than girls [2]. ASD is associated with brain and/or systematic physiological abnormalities including metabolic, mitochondrial and immune disorders as well as high levels of oxidative stress [3]. Metabolic disorders are of particularly clinical relevance, as many are amenable to targeted and safe, well tolerated treatments [4,5].

Understanding these underlying disorders can assist in the development of objective biomarkers to assist with diagnosis, identify subgroups and screen for the best candidates for the most effective treatment [6]. These biological abnormalities have been leveraged to develop objective biomarkers, however despite promising advances, no single biomarker has yet demonstrated high enough sensitivity, specificity and clinical utility to be implemented in clinic practice [7].

Disorders of mitochondrial function may affect a substantial number of individuals with ASD [8]. For example, studies have found that up to 80% of children with ASD were found to have electron transport chain (ETC) complex deficits [9,10]. Interestingly, mitochondria abnormalities associated with ASD are typically non-classical in nature [11] with studies ETC complex activity and mitochondrial respiration found to be increased rather than decreased as is found in classic mitochondrial disease [8].

Using the Seahorse 96 XF high-throughput respiratory analyzer our group found that the lymphoblastic cell lines (LCLs) from about one-third of children with ASD manifested elevated respiratory rates (about 200% of controls) and were more vulnerable to physiological stress. Reserve capacity (RC), a functional indicator of mitochondrial health and bioenergetic resilience, sharply declined in these ASD LCLs when challenged with physiological stress as compared to other LCLs [8]. These physiologically vulnerable cell lines are called AD-A (ASD with abnormal mitochondrial function). Mitochondria from the remaining ASD LCLs were essential equivalent to controls and are classified as AD-N (ASD with normal mitochondrial function). We have replicated these results in LCLs in seven additional studies and have demonstrated that this pattern of respiratory abnormalities is also manifested in fresh peripheral blood mononuclear cells (PBMCs) from individuals with ASD, especially children with neurodevelopmental regression [8]. In our investigations of the modulator mechanisms driving the respiratory abnormalities, we observed that the AD-A LCLs, as compared to AD-N LCLs, failed to upregulate key regulators of mitochondrial biogenesis and homeostasis—specially PINK1, MFN2, SIRT1, SIRT3 DNM1L, HIF1z and PGC1a [12]. Notably, this increase in mitochondria respiration could be mitigated by rapamycin, implicating the mTOR pathway.

To obtain further insight into the molecular mechanisms which drive the mitochondrial phenotype in the AD-A LCLs, this study used RNAseq mRNA and miRNA profiling in the LCLs to examine whether the differentially expressed genes are related to the mTOR or cellular and mitochondrial stress pathways previously identified. Overall, the findings from this study offer mechanistic insights into a distinct ASD endophenotype and the promise of developing biomarkers to identify this endophenotype.

## 2. Materials and Methods

### 2.1. Lyphobalstopid Cell Lines

Materials, reagents and details regarding LCL source and culture techniques have been outlined in our previous work [13]. LCLs were obtained from the Autism Genetic Resource Exchange (AGRE; Los Angeles, CA, USA). Details of the LCLs are presented in Supplement Table S1. Age was not significantly difference across groups as measured by paired *t*-test. These LCLs were used in a previous study examining bioenergetics [14].

### 2.2. Sequening and Bioinformatics

Library construction, sequencing and initial bioinformatics analysis has been described in our previous studies [13]. Differential expression of the two groups was performed using the DESeq2 R package (1.20.0), which uses a model based on the negative binomial distribution. The *p* value of mRNA differential analysis was corrected for the false discovery rate (FDR) using the Benjamini and Hochberg’s approach. Genes with an FDR < 0.05 were regarded as differential expressed genes.

### 2.3. Target Prediction and Enrichment Analysis

Differentially expressed genes (DEGs) were selected to test for functional enrichment analysis using IPA software. Differentially expressed miRNAs were selected to determine the specific genes and pathways that were targeted by the miRNAs using miRPath v4.0 using TarBase v8.0, annotation miRBase-v22.1 Hallmark MSigDB gene sets classic analysis method and FDR corrected *p* ≤ 0.05. To determine if particular miRNAs were associated with specific genes, miRWalk2.0 was used with a threshold of 1.0 [15]. The miRWalk2.0 platform performs a robust meta-analytic integration of predicted miRNA–mRNA interactions by aggregating data from 13 independent target prediction algorithms. This multi-source consensus framework enhances the stringency of target identification and minimizes false-positive rates by leveraging algorithmic complementarity and cross-validation across diverse computational methodologies [16,17]. For pathway visualization of miRNA, miRTarget Link 2.0 was using validated and predicted miRNA targets with a minimum of four shared targets [18].

### 2.4. Relationship to Genes Previous Identifed Differentiating AD-A and AD-N

Several studies have identified gene expression differences in LCLs between the AD-A and AD-N groups. These include *UCP2*, *mTOR*, *AMPK*, *PINK1*, *PTEN*, *MFN2*, *PTEN*, *AKT1*, *SOD2*, *SIRT1*, *SIRT3*, *DNM1L*, *HIF1A*, *PPARGC1A*. In our previous studies comparing AD-N and AD-A we found that the mTOR pathway was highly involved in modulation of respiration in the AD-A groups. Thus, we examined the relation of miRNAs to core mTOR genes, including *MTOR*, *RPTOR*, *RICTOR*, *mST8*, *DEPTOR*, *PRAS40*, *mSIN1* as well as upstream regulators *AMPK* (*PRKAA1*, *PRKAA2*, *PRKAB1*, *PRKAB2*, *PRKAG1*, *PRKAG2*, *PRKAG3*), *LKB1*, *AKT* (*AKT1*, *SKT2*, *AKT3*), *PTEN*, *RHEB*, *TSC1*, *TSC2*, *I3K* (*PIK3CA*, *PIK3CB*, *PIK3CD*, *PIK3R1*, *PIK3R2*, *PIK3R3*). We also examine the genes identified and verified in our previous miRNA manuscript differentiating ASD and siblings controls. These include *AUTS2*, *FMR1*, *IL27*, *FOXP1*, *NTN1*, *NCAM2*, *GABRA4*. In our previous study we also found that Calmodulin Kinase II was differentially expressed and correlated with ASD severity, so we examined *CAMK2A*, *CAMK2B*, *CAMKK2*.

## 3. Results

### 3.1. Identification of Differentially Expressed Genes (DEGs)

**Figure 1:**
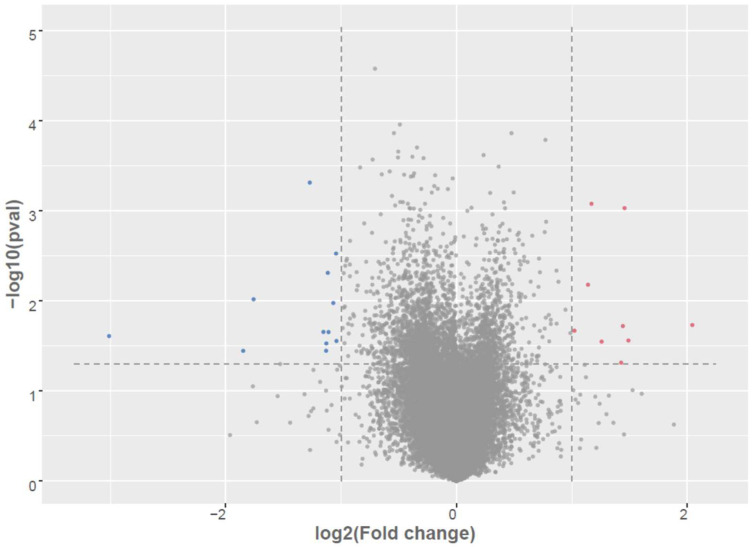
Differential expression of mRNAs in LCLs groups. Volcano plot of differentially expressed between AD-A and AD-N. Red dots are up-regulated, Blue dots are down-regulated; Grey dots no change; To determine significant genes (red and blue color dots), the *p*-value cut-off was set to 0.05 and fold change of 1.0.

Table 1 summarizes 24 DEGs between the AD-A and AD-N groups identified through RNA-seq analysis using a significance threshold of *p*  <  0.05 and an absolute log_2_ fold change > 1.0 (See Figure 1). Among these, 14 transcripts were significantly downregulated (i.e., elevated in AD-A relative to AD-N), while 10 exhibited upregulation (i.e., elevated in AD-N). Genes upregulated in the AD-N cohort include mitochondrial ribosomal protein MRPL41 and the nuclear RNA processing gene RPP25L, suggesting perturbed mRNA maturation dynamics. Additionally, transcripts linked to immunological function (e.g., HLA-DQA2, HEXIM1, PNMA1) and oncogenic pathways (HEXIM1, FRAT2, PNMA1, HMGA1) were enriched.

Conversely, the AD-A group demonstrated upregulation of several transcripts implicated in tumorigenesis (CAB39), intracellular signaling (CAB39), autoimmunity (IFIT3), and mTOR signaling (DEPTOR). Genes involved in transcriptional regulation (TCEAL8, DDX21, CHD3, HIST2H2BE) and phospholipid metabolism (LYPLA1) were also significantly elevated. Notably, none of these DEGs are curated within the SFARI gene database, indicating a potential divergence from established ASD-related gene sets.

**Figure 2:**
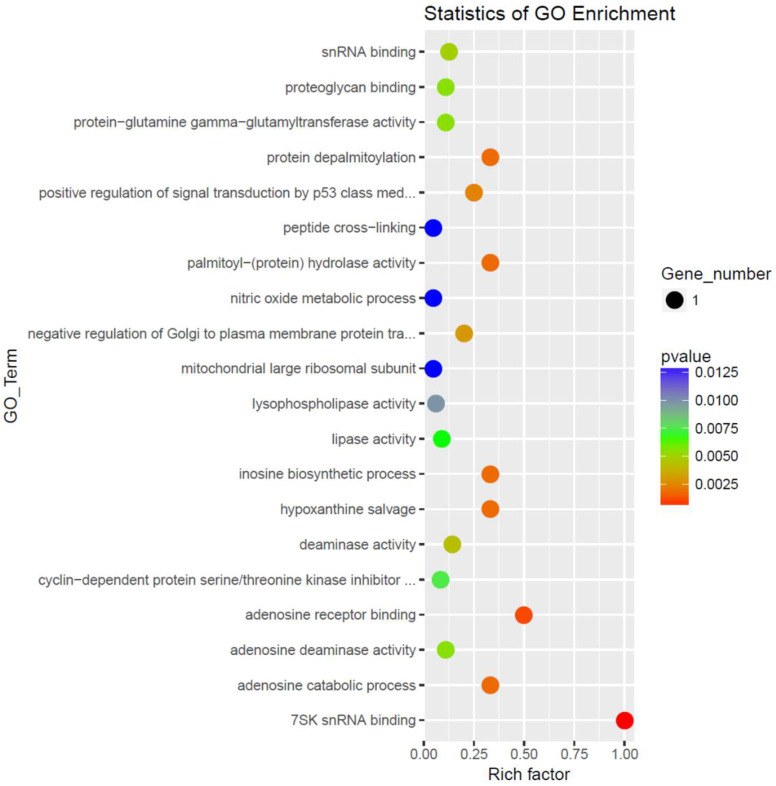
Enrichment analysis of the differentially expressed mRNAs by IPA software. The top 20 enriched GO functional processes of the target genes of differentially expressed mRNAs. Red = activated; Blue = Inhibited.

GO enchainment analysis (Figure 2) shows several processes with high rich factor and statistical significance including 75K snRNA binding, adenosine receptor binding and catabolic processes, palmitoyl-protein hydrolase and protein depalmitoylation activity and positive regulator of signal transduction of p53. Significantly inhibited pathways were of peptide cross-linking, nitric oxide metabolic process and mitochondrial large ribosomal subunit.

To elucidate the biological relevance of differentially expressed genes, we examined their association with Gene Ontology (GO) biological processes, focusing on those reaching statistical significance (*p* < 0.05), as summarized in Table 2. Among the genes enriched in the AD-N subgroup, HEXIM1 and MRPL41 emerged as consistently implicated in distinct molecular pathways. HEXIM1 was associated with cyclin-dependent protein serine/threonine kinase activity and positive regulation of p53 class mediators while MRPL41 was related to gene translation in the mitochondria. CECR1, DDX21, F13A1, LYPLA1 were consistently related to processes increased in the AD-A group. CECR1 GO processes all points to adenosine catabolism and salvage. DDX21 GO processes all point to RNA metabolism. F13A1 GO processes all point to healing processes. LYPLA1 GO processes point to lipoprotein modifications and membrane signaling dynamics. FRAT2, HEXIM1, MRPL41, CECR1, DDX21, RPP25L had genes with both increased and decreased differential expression between groups.

### 3.2. Identification of Differentially Expressed miRNAs (DEMs)

Annotation of total and unique miRNA species across AD-A and AD-N revealed comparable distribution profiles, as illustrated by the pie charts in Supplementary Figure S1A,B. Analysis of read length distributions across these groups demonstrated a pronounced enrichment around 22 nucleotides, with the majority of high-confidence reads falling within the 21–24 nt range (Supplementary Figure S2), consistent with canonical miRNA length signatures.

**Figure 3:**
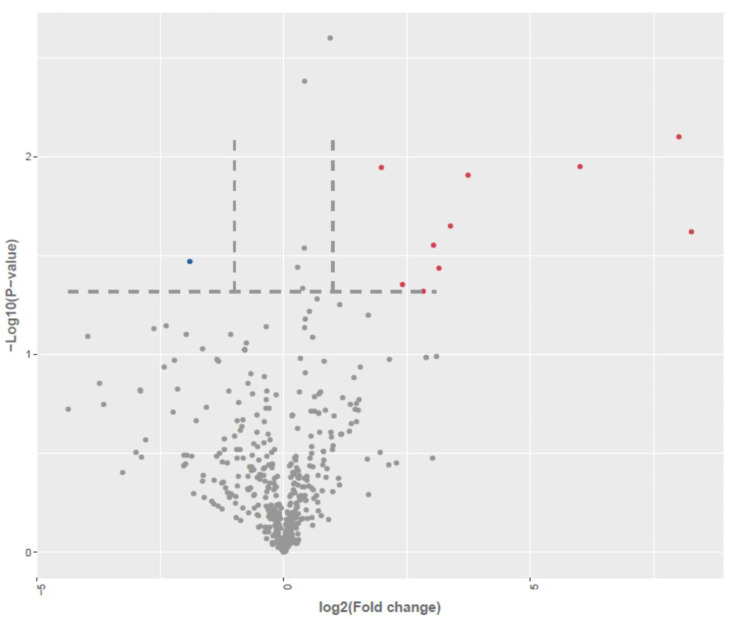
Differential expression of miRNA in LCLs groups by small RNA-seq analysis (miRNA seq). Volcano plot of differentially expressed between AD-A and AD-N. Red dots are up-regulated, Blue dots are down-regulated; Grey dots no change; To determine significant genes (red and blue color dots), the *p*-value cut-off was set to 0.05 and fold change of 1.0.

Differential expression analysis, visualized through volcano plots (Figure 3), identified a subset of both annotated and novel miRNAs meeting stringent thresholds of |log_2_ fold change| > 1.0 and *p*  ≤  0.05. A comparative analysis between AD-N and AD-A samples revealed 19 significantly dysregulated miRNAs, including 18 upregulated and 1 downregulated transcripts out of 525 detected miRNA species. The 1 downregulated transcript is provisional, so it was not included in further analyses.

These differentially expressed miRNAs expressed in brain, along with their fold-change values and statistical significance, are detailed in Table 3 [19]. miRNAs that are heavily expressed in brain are bolded. Three of the miRNAs (bta-mir-1246-p3_1ss2CT, hsa-miR-3687, hsa-miR-1254) are not found in the central nervous system are not shown. Heatmap visualization given in Supplementary Figure S3 illustrates expression dynamics across samples, using a two-color gradient to depict relative abundance—ranging from red (upregulation) to blue (downregulation). Integrative analysis revealed that a subset of dysregulated miRNAs—namely hsa-miR-1273h-3p, hsa-miR-197-3p, hsa-miR-199a-5p, hsa-miR-204-5p, hsa-miR-874-5p, hsa-miR-100, hsa-miR-941 and hsa-miR-769-5p—are predicted to target mRNAs previously identified as differentially expressed, suggesting coordinated post-transcriptional regulatory mechanisms driving disease-specific transcriptomic remodeling. mRNA targets that have been empirically validated are bolded with the peer-reviewed validation studies given. 

Several of the miRNAs identified were found to target the mRNAs identified above. hsa-miR-199a-5p targets F13A1, hsa-miR-204-5p targets HEXIM1, FAM110A and HMGA1, hsa-miR-874-5p targets PNMA1, hsa-miR-100-5p targets IFIT3, hsa-miR-941 targets DDX21, CHD3 and hsa-miR-769-5p targets CHD3.

#### 3.2.1. Enrichment Analysis

**Figure 4:**
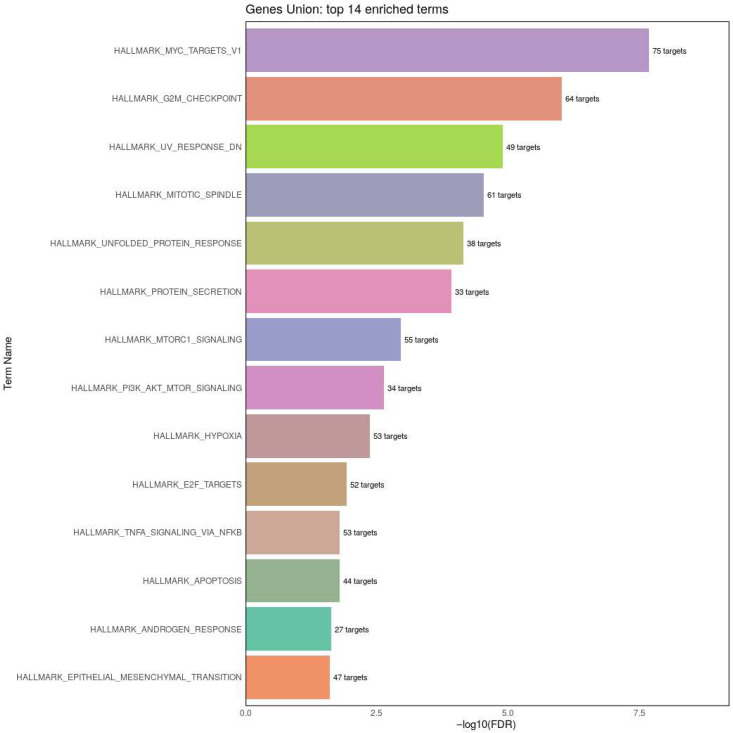
Enrichment analysis of the differentially expressed miRNAs.

The graph in Figure 4 represents the union of the target genes of the differentially dysregulated miRNAs. mTOR signaling (PI3K/AKT/mTOR signaling, mTORC1 signaling) is targeted by these miRNAs with other targets including regulation of cell growth and division (G2M checkpoint, Mitotic Spindle, Myc targets), gene expression (E2F targets), immune regulation (NF-ĸB signaling) and cellular stress (unfolding protein response).

**Figure 5:**
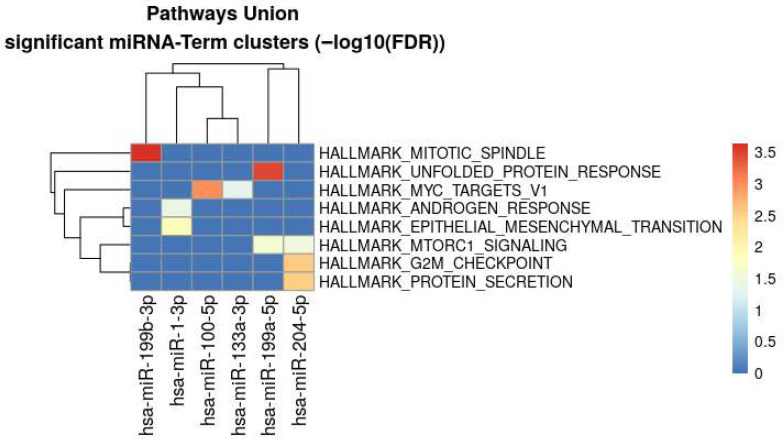
Integrative pathway analysis demonstrated convergence on mTOR and other cell growth pathways.

To assess whether the differentially expressed miRNAs converge on common molecular pathways, we utilized miRPath v4.0 to perform integrative pathway analysis based on the union of miRNAs targeting both experimentally identified mRNAs and computationally predicted gene targets [52] (See Figure 5).

#### 3.2.2. Pathway Visualization

**Figure 6:**
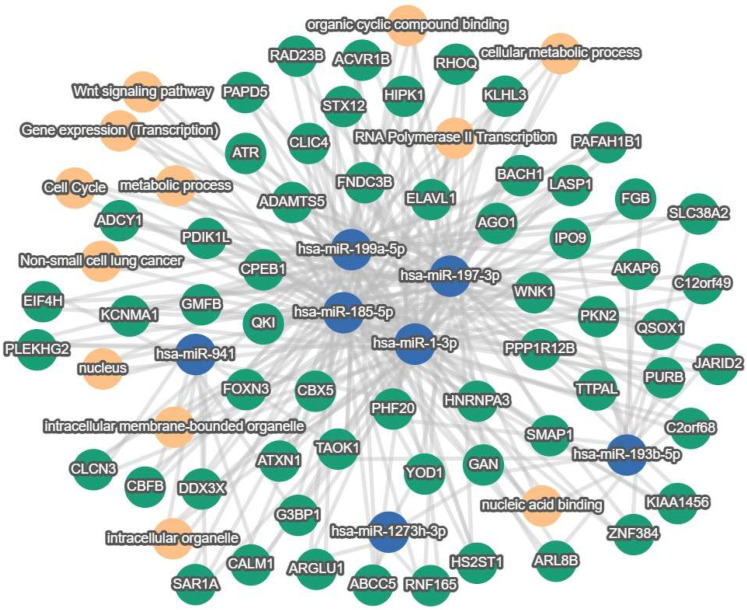
Pathway visualization analysis showing the mutual connections (threshold 4) for identified differentially expressed miRNAs. Several miRNAs for a central hub to genes involved in nervous system development, cell growth and regulation of RNA. Blue circles are miRNAs, green circles are mRNAs and orange circles are cellular processes, pathways and related disorders.

Pathway visualization demonstrated that has-miR-197-3p, has-miR-185-5p, has-miR-199-5p and has-miR-1-3p were highly interconnected and the hub for many common genes and cellular processes (Figure 6). The cellular processes include metabolic processes, organic cyclic compound binding, intracellular membrane-bound organelles, RNA Polymerase II Transcription, gene expression and transcription and nucleic acid binding. The genes linked to these central miRNAs included cell growth regulatory, RNA regulation and neuronal genes. Genes related to the nervous system including ADCY1, a enzymes important in synaptic plasticity, memory formation, learning, and adaptation to sensory stimuli involved in cognition and emotion, found in the hippocampus, cortex, thalamus, and cerebellum [53], PAFAH1B1 (LIS1) which is a regulator of the microtubule-based motor protein dynein which ensures accurate neuronal migration during embryonic brain [54], GMFB, a highly conserved neurotrophic protein pivotal in glial and neuronal differentiation and maturation [55], and KCNMA1 which encodes a potassium channel critical in neurotransmitter release and neuronal excitability and has been implicated in multiple neurological disorders [56]. Gene involved in cell growth and replication include HNRNPA3 which modulates telomere dynamics [57], and PAFAH1B1 (LIS1) which affects microtubule organization, affecting cell division [58], PDIK1L, a serine/threonine protein kinase that plays a key regulatory role in cell cycle progression and translational initiation [59]. Genes involved in RNA regulation, including PAPD5 which is involved in RNA surveillance, turnover, and homeostasis [60], HNRNPA3 which is involved in mRNA splicing, cytoplasmic RNA trafficking [61], QKI which is an RNA-binding protein which has an essential role in alternative splicing, mRNA stability, translation, and cellular differentiation [62], ELAVL1 a RNA-binding protein that stabilizes and regulates the translation of target mRNAs which is primary expressed in the nervous system [63], PAPD5, a noncanonical poly (A) polymerase that plays a central role in RNA surveillance, turnover, and quality control [60].

#### 3.2.3. Discriminant Analysis

**Figure 7:**
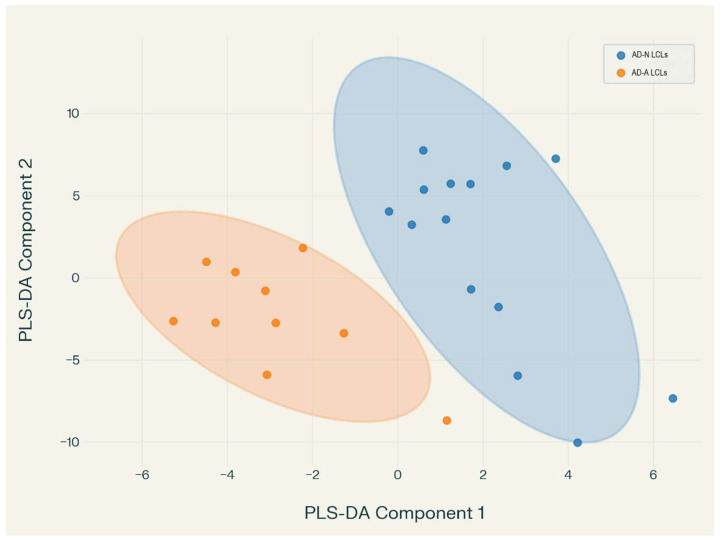
Values for the first two components for the individual LCLs divided into there two groups with 95% confidence oval for the groups.

To determine if miRNA, as a group, were different in the AN-A and AD-N LCLs, partial least squares discriminant analysis was used to produce a function which discriminated between AD-A and AD-N LCLs. The function demonstrated 100% accuracy. The first two components explained at least 80% of the variance while the first three components explained over 90% of the variance. Figure 7 demonstrates the values of individual LCLs in each group for the first two factors of the discriminant analysis with the 95% confidence oval. The two groups are clearly separated using these two factors.

Several miRNA were significant in the discriminant function including hsa-miR-1273h-3p, hsa-miR-197-3p, hsa-miR-1-3p, hsa-miR-615-3p_R-1, hsa-miR-199a-5p, hsa-miR-185-5p, hsa-miR-193b-5p. Both hsa-miR-1-3p and hsa-miR-199a-5p overlap with the above findings. Several other miRNAs are confirmed mTOR targets with miR-197-3p [64,65], miR-185-5p [66] and miR-193b-5p [67,68] suppressing mTOR and miR-615-3p_R-1 indirectly activating mTOR [69,70]. miRPath v4.0 miRNA-centric gene and pathway union identified hypothesized pathways including mTOR as well as other pathways previously identified in the differential expression analysis above (See Figures S5 and S6).

## 4. Discussion

We report the identification of two biologically distinct subgroups of autism spectrum disorder (ASD) based on mitochondrial functional phenotypes in lymphoblastoid cell lines (LCLs): AD-A (ASD with aberrant mitochondrial function characterized by elevated respiration and stress sensitivity) and AD-N (ASD with normative mitochondrial function). To elucidate molecular mechanisms underlying these phenotypes, we conducted integrated transcriptomic profiling—encompassing both mRNA and miRNA sequencing—followed by differential expression analysis, functional enrichment, and pathway interrogation, with a particular focus on mTOR and mitochondrial stress-related genes previously implicated in these ASD subtypes.

RNA-seq analysis revealed 24 mRNAs differentially expressed between AD-A and AD-N groups (*p*  <  0.05, |log_2_FC| > 1), with 14 transcripts upregulated in AD-A and 10 in AD-N. Upregulated transcripts in AD-N included MRPL41 and RPP25L, implicated in mitochondrial and nuclear RNA processing, respectively, as well as immune-related and oncogenic transcripts (HLA-DQA2, HEXIM1, PNMA1). In contrast, AD-A LCLs exhibited increased expression of genes associated with intracellular signaling (CAB39), autoimmune processes (IFIT3), and mTOR pathway regulation (DEPTOR), as well as genes involved in transcriptional control (TCEAL8, CHD3, DDX21) and lipid metabolism (LYPLA1). Gene Ontology (GO) enrichment analysis highlighted perturbations in snRNA binding, purinergic signaling, p53 regulation, and depalmitoylation processes.

Out of 525 detected miRNAs, 18 were upregulated and 1 was downregulated in AD-N as compared to AD-A. Notably, several differentially expressed miRNAs, including hsa-miR-1273h-3p, hsa-miR-197-3p, hsa-miR-199a-5p, hsa-miR-204-5p, hsa-miR-874-5p, hsa-miR-100-5p, hsa-miR-941, and hsa-miR-769-5p, were predicted to target both the genes identified in the mRNA analysis and those previously implicated in ASD, such as the mTOR signaling cascade and Calmodulin Kinase II.

Pathway enrichment analysis of miRNA revealed overrepresentation of pathways involved in these hypothesized pathways as well as other pathways, particularly cell growth and division. Perhaps this is not surprising given the role of mTOR is regulating cell growth and mitotic progression [71]. mTOR also regulates mitochondrial function [72], which was found to be abnormal in a subset of the LCLs we examined, as well as is regulated by cellular energetics [73], being central to mitochondrial regulation.

The results show a miRNA-mRNA network potentially regulating mitochondrial function in ASD, particularly the mTOR, PI3K/AKT, and autophagy pathways previously associated with mitochondrial regulation, immune response, and neuronal health. These findings provide further support for subtype-specific molecular signatures in ASD and suggest that differential miRNA expression may contribute to the heterogeneity of ASD severity through modulation of convergent neurodevelopmental and metabolic pathways.

Of particular note, the mechanistic target of rapamycin (mTOR) emerged as a central node in the transcriptomic network. mTOR exists in two functionally distinct complexes: mTORC1, which governs protein synthesis, mitochondrial metabolism, and autophagy, and mTORC2, which modulates cytoskeletal dynamics and cell survival. Hyperactivation of mTORC1—a phenomenon documented in a subset of ASD individuals—impairs mitophagy, elevates mitochondrial ROS, synaptic pruning and disrupts ATP homeostasis, culminating in synaptic and dendritic pathologies linked to core ASD features [20,27,28,29].

CaMKII also has a critical role in post-synaptic neuronal function where it modulates long-term potentiation and NMDA-dependent synaptic plasticity [74,75]. CaMKII has an important role in regulation of NMDA receptors, resulting in neuronal excitability through glutamate transmission [76]. This may be significant as dysregulation of long-term potentiation [77] and glutamate regulation [78] has been implicated in psychiatric disorders. Mutations in CaMKIIα and CaMKIIβ are association with intellectual disability [79] and ASD behaviors [80].

Our previous study examining miRNA which distinguished ASD LCLs from controls identified two candidate miRNAs, hsa-miR-181a-5p and hsa-miR-320a [13], which are highly expressed in brain and spinal cord [81] and they have been identified as dysregulated in previous ASD studies [82,83,84]. Interestingly, both miR-181a-5p [85,86] and miR-320a [87] target AKT3, a key regulator of the PI3K-AKT-mTOR [88] and PTEN/Akt/TGF-β1 signaling pathway [89], two pathways which overlap the pathways identified in this study and are known to be involved in ASD.

## 5. Conclusions

This study underscores the molecular heterogeneity of ASD by identifying a distinct ASD subgroup characterized by abnormal mitochondrial function (AD-A) and unique miRNA–mRNA expression profiles. The enrichment of transcripts involved in mTOR signaling, cellular stress responses, and mitochondrial regulation within this subgroup reveals convergent pathways that plausibly drive its metabolic phenotype. These molecular perturbations offer more than mechanistic insight: they provide concrete, actionable signatures that could serve as biomarkers for stratification and tailoring interventions. Importantly, the delineation of ASD subtypes based on mitochondrial and transcriptional dysregulation offers a critical step toward precision diagnostics and paves the way for pathway-targeted therapies tailored to metabolically vulnerable individuals with ASD.

## Figures and Tables

**Table 1:** Top differentially expressed mRNAs between AD-A and AD-N LCLs.

**Up Regulated in AD-N (LCLs from Children with Autism with Normal Mitochondrial Function). **			
**mRNA**	**log2(FC)**	**−log10(p)**	**Gene Name**
MRPL41	2.04	1.73	Mitochondrial ribosomal protein l41
HLA-DQA2	1.49	1.56	Major histocompatibility complex, class ii, dq alpha-2
GAPDHP1	1.46	3.03	Glyceraldehyde-3-phosphate dehydrogenase pseudogene 1
RPP25L	1.44	1.72	Ribonuclease P/MRP Subunit p25
SNRPFP2	1.43	1.31	Small nuclear ribonucleoprotein polypeptide F pseudogene 2
HEXIM1	1.26	1.55	Hexamethylene bis acetamide-inducible protein 1
FRAT2	1.17	3.08	Frequently rearranged in advanced t-cell lymphomas 2
FAM110A	1.14	2.18	Family with sequence similarity 110
PNMA1	1.11	1.29	Paraneoplastic antigen ma1
HMGA1	1.02	1.67	High mobility group at-hook 1
**Down Regulated in AD-N (LCLs from Children with Autism with Normal Mitochondrial Function)**			
**mRNA** **ID**	**log2(FC)**	**−log10(p)**	**Gene Name**
CTD-2287O16.1	−3.01	1.61	Pseudogene
CAB39	−1.85	1.45	Calcium-binding protein 39
RP11-603J24.17	−1.76	2.02	Antisense gene
IFIT3	−1.53	1.30	Interferon-induced protein with tetratricopeptide repeats 3
CECR1	−1.27	3.31	Adenosine deaminase 2
DEPTOR	−1.15	1.65	Dep domain-containing protein 6
F13A1	−1.13	1.45	Factor xiii, a1 subunit
TCEAL8	−1.13	1.53	Transcription elongation factor a like 8
LYPLA1	−1.12	2.31	Lysophospholipase i
IQUB	−1.11	1.65	Iq motif- and ubiquitin domain-containing protein
FAM103A2P	−1.07	1.98	RNA guanine-7 methyltransferase activating subunit like
DDX21	−1.04	2.52	Dexd-BOX HELICASE 21
CHD3	−1.04	1.55	Chromodomain helicase dna-binding protein 3
HIST2H2BE	−1.01	1.28	Histone gene cluster 2, h2b histone family, member e

**Table 2:** GO processes related to differentially expressed mRNA genes.

Gene	*p*-Value	GO Functional Process
**Upregulated Pathways in ASD LCL with Normal Mitochondrial Function**		
		Biological Process
Hexamethylene bis acetamide-inducible protein 1	0.002	Positive regulation of signal transduction by p53 class mediator
	0.01	Negative regulation of cyclin-dependent protein serine/threonine kinase activity
		Molecular function
	0.01	Snrna binding
	0.01	Cyclin-dependent protein serine/threonine kinase inhibitor activity
		Biological Process
Mitochondrial ribosomal protein l41	0.05	Mitochondrial translational termination
	0.05	Mitochondrial translational elongation
	0.05	Mitochondrial translational initiation
		Cellular component
	0.01	Mitochondrial large ribosomal subunit
**Mixed Regulation**		
	0.03	Biological Process
Frequently rearranged in advanced t-cell lymphomas 2 (up)		Multicellular organismal development
Adenosine deaminase 2 (down)		
		Molecular Function
Dexd-BOX HELICASE 21 (down)	0.03	Poly(A) RNA binding
Mitochondrial ribosomal protein l41 (up)		
Ribonuclease P/MRP Subunit p25 (up)		
**Down Regulated in ASD LCL with Normal Mitochondrial Function**		
		Biological Process
Adenosine deaminase 2	0.001	adenosine catabolic process
	0.001	hypoxanthine salvage
	0.001	inosine biosynthetic process
		Molecular Function
	0.001	Adenosine receptor binding
	0.01	Deaminase activity
	0.01	Proteoglycan binding
	0.01	Adenosine deaminase activity
		Biological Process
Dexd-BOX HELICASE 21	0.02	Response to exogenous dsrna
	0.03	RNA secondary structure unwinding
		Molecular Function
	0.00	7SK snrna binding
	0.01	Snorna binding
	0.02	rRNA binding
	0.03	Double-stranded RNA binding
	0.04	ATP-dependent RNA helicase activity
	0.05	Helicase activity
		Biological Process
Factor xiii, a1 subunit	0.01	Peptide cross-linking
	0.05	Platelet degranulation
	0.05	Wound healing
		Cellular component
	0.03	Platelet alpha granule lumen
		Molecular Function
	0.01	Protein-glutamine γ-glutamyltransferase activity
		Biological Process
Lysophospholipase I	0.00	Protein depalmitoylation
	0.00	Negative regulation of Golgi to plasma membrane protein transport
	0.01	Nitric oxide metabolic process
	0.01	Regulation of nitric-oxide synthase activity
	0.04	Fatty acid metabolic process
		Molecular Function
	0.00	Palmitoyl-(protein) hydrolase activity
	0.01	Lipase activity
	0.01	Lysophospholipase activity

**Table 3:** Top differentially expressed miRNAs between AD-A and AD-N LCLs along with empirical and bioinformatic associations with proposed and experimental identified genes of interest. miRNAs heavily expressed in the brain are bolded. mRNAs that are validated targets are bolded.

Up Regulated in AD-N (LCLs from Children with Autism with Normal Mitochondrial Function)				
**miRNA ID**	**log2(FC)**	**−log10(p)**	**Predicted**	**mTOR Pathway**
**hsa-miR-133a-3p**	8.27	1.62	**UCP2** [20,21], **AKT1** [21,22,23], **PTEN** [21]	**mTOR** [21] **AMPK** [24,25]
hsa-miR-1-3p	8.02	2.10	**UCP2** [20,26]**, PTEN** [27]**, MFN2** [27]**, AKT1** [26]**, SOD2** [28]**, SIRT1** [29]**, PPARGC1A** [29]	
**hsa-miR-126-3p**	6.01	1.95	HIF1A, PPARGC1A, SIRT1, **SOD2** [30], **AKT1** [31], **PTEN** [32]	**mTOR** [33]
**hsa-miR-199a-5p**	3.74	1.91	**PTEN** [27]**, SOD2** [34], **HIF1A** [35], MFN2, DNM1L, AUTS2, FMR1, IL27, CAMK2A, CAMKK2	**mTOR** [36], TSC1, RHEB, AKT3, PIK3R3
**hsa-miR-204-5p**	3.38	1.65	**PTEN** [37,38], DNM1L, SIRT3, HIF1A, CAMK2A	**AKT1** [38] AKT3, **PIK3R3** [39]
hsa-miR-874-5p	3.15	1.43	**PTEN** [40], CAMK2B, MFN2, AKT1S1, DNM1L, HIF1A, CREB1, PPARGC1A,	**mTOR** [41], TSC1/2, AKT2, **PIK3CD** [42]
**hsa-miR-100-5p**	3.04	1.55	**PTEN** [43]**, SOD2** [44]**, HIF1A** [44,45]**,** CREB1,	**mTOR** [46]**, PIK3CA** [47] **PIK3CB** [47]
hsa-miR-941	1.34	1.54	SOD2, PPARGC1A, **HIF1A** [48]**, PTEN** [48]	TSC1, AKT2/3, PIK3R3, **PIK3CA,B** [48]
**hsa-miR-769-5p**	1.22	1.44	HIF1A, AUTS2	AKT3
**hsa-miR-199b-3p**	2.83	1.32	**SIRT1** [49]**, HIF1A** [49]	**mTOR** [50]**, AKT1** [51]

Bold for the miRNA that are related are found in brain. Bold for mRNA are those that have been validated.

## Data Availability

Data is available upon request.

## Supplementary Materials

The following are available online at https://www.mdpi.com/article/10.3390/genes16091065/s1, Table S1: Lymphoblastoid Cell Lines (LCLs) used in this study; Figure S1: Distribution of small RNA type, Figure S2: Length Distribution of miRNAs; Figure S3: Heat Map for AD-N vs. AD-A; Figure S4: miRNA heatmap; Figure S5: Gene enrichment using miRNAs identified by PLSDA; Figure S6: Pathway enrichment using miRNAs identified by PLSDA.
