# Supplementary material for: Transcriptomic Signatures of Mitochondrial Dysfunction in Autism: Integrated mRNA and microRNA Profiling
Source: Genes (Basel). 2025 Sep 10;16(9):1065. doi: 10.3390/genes16091065 (PMC12469284; doi:10.3390/genes16091065)
Supplement: Supplementary file 1 [file genes-16-01065-s001.zip › Supplement Table S1.pdf]

Supplement Table S1. Lymphoblastoid Cell Lines (LCLs) used in this study. LCLs are from males with Autistic Disorder (AD) and the groups were based upon mitochondrial acivity as AD-Normal (N) and AD-Abnormal (A). Average age of each group is provided. There is no significant age difference between groups (p=0.31).

AD-N		AD-A	
**ID**	**Age**	**ID**	**Age**
**4363**	**4**	**3540**	**3**
**8022**	**5**	**8495**	**4**
**10054**	**6**	**3620**	**7**
**7439**	**7**	**8594**	**7**
**10618**	**7**	**3110**	**7**
**8367**	**7**	**9713**	**7**
**9650**	**7**	**14441**	**7**
**0775**	**8**	**2591**	**11**
**4757**	**10**	**16499**	**11**
**3497**	**10**	**2746**	**13**
**38988**	**12**		
**2942**	12		
**3563**	**12**		
**1129**	13		
**4349**	**17**		
	9.1 (3.4)		7.7 (3.0)
