# Supplementary material for: Transcriptomic Signatures of Mitochondrial Dysfunction in Autism: Integrated mRNA and microRNA Profiling
Source: Genes (Basel). 2025 Sep 10;16(9):1065. doi: 10.3390/genes16091065 (PMC12469284; doi:10.3390/genes16091065)
Supplement: Supplementary file 1 [file genes-16-01065-s001.zip › Supplementary Figure S1.pdf]

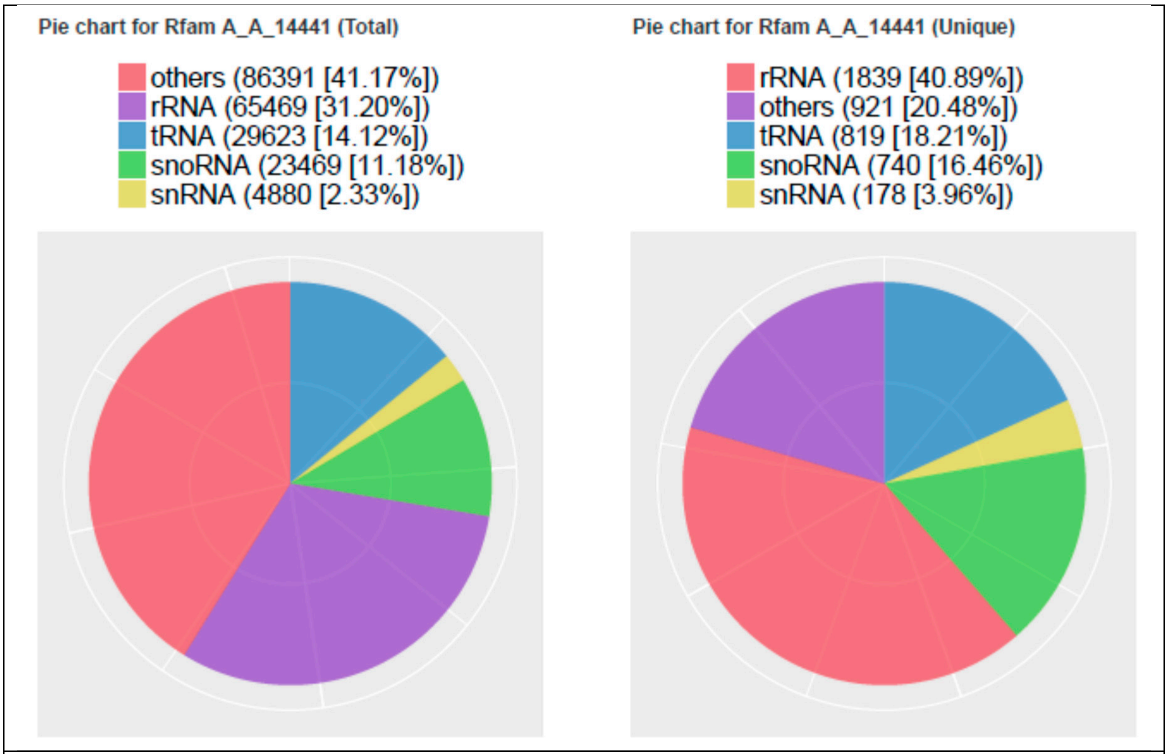
Pie chart for Rfam A_A_14441 (Total)
others (86391 [41.17%])
rRNA (65469 [31.20%])
tRNA (29623 [14.12%])
snoRNA (23469 [11.18%])
snRNA (4880 [2.33%])
Pie chart for Rfam A_A_14441 (Unique)
rRNA (1839 [40.89%])
others (921 [20.48%])
tRNA (819 [18.21%])
snoRNA (740 [16.46%])
snRNA (178 [3.96%])

(A)

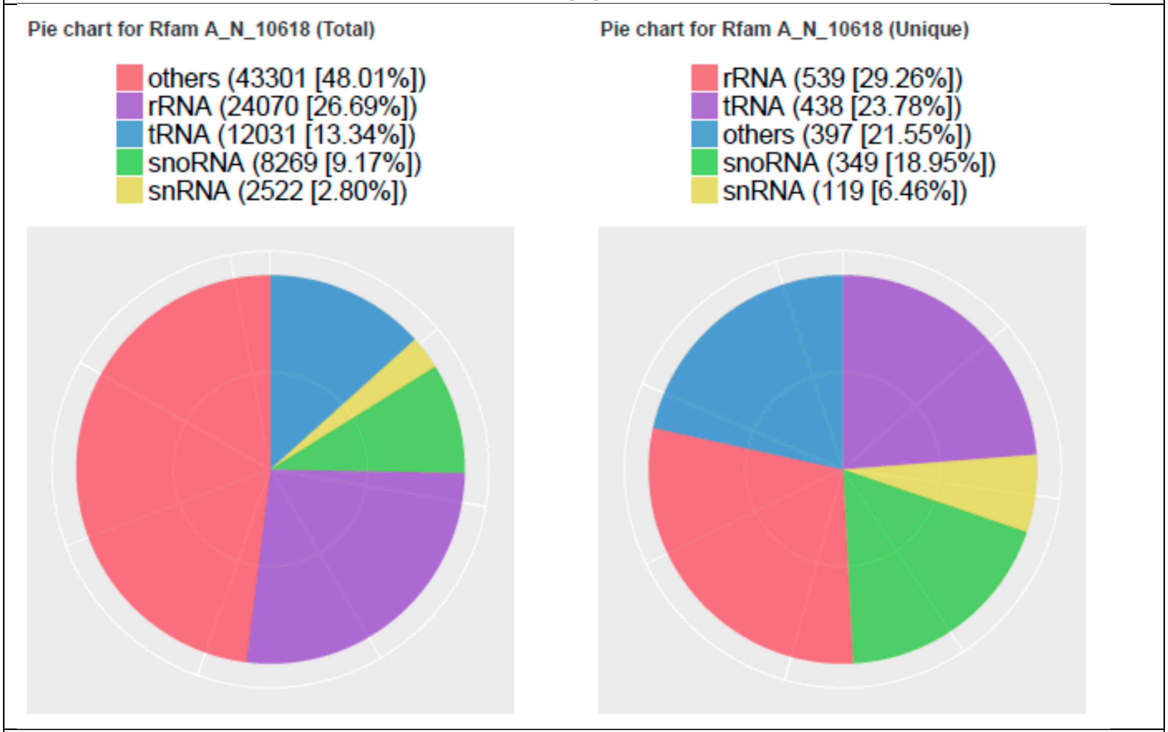
Pie chart for Rfam A_N_10618 (Total)
others (43301 [48.01%])
rRNA (24070 [26.69%])
tRNA (12031 [13.34%])
snoRNA (8269 [9.17%])
snRNA (2522 [2.80%])
Pie chart for Rfam A_N_10618 (Unique)
rRNA (539 [29.26%])
tRNA (438 [23.78%])
others (397 [21.55%])
snoRNA (349 [18.95%])
snRNA (119 [6.46%])

(B)
