# Supplementary material for: Transcriptomic Signatures of Mitochondrial Dysfunction in Autism: Integrated mRNA and microRNA Profiling
Source: Genes (Basel). 2025 Sep 10;16(9):1065. doi: 10.3390/genes16091065 (PMC12469284; doi:10.3390/genes16091065)
Supplement: Supplementary file 1 [file genes-16-01065-s001.zip › Supplementary Figure S2.pdf]

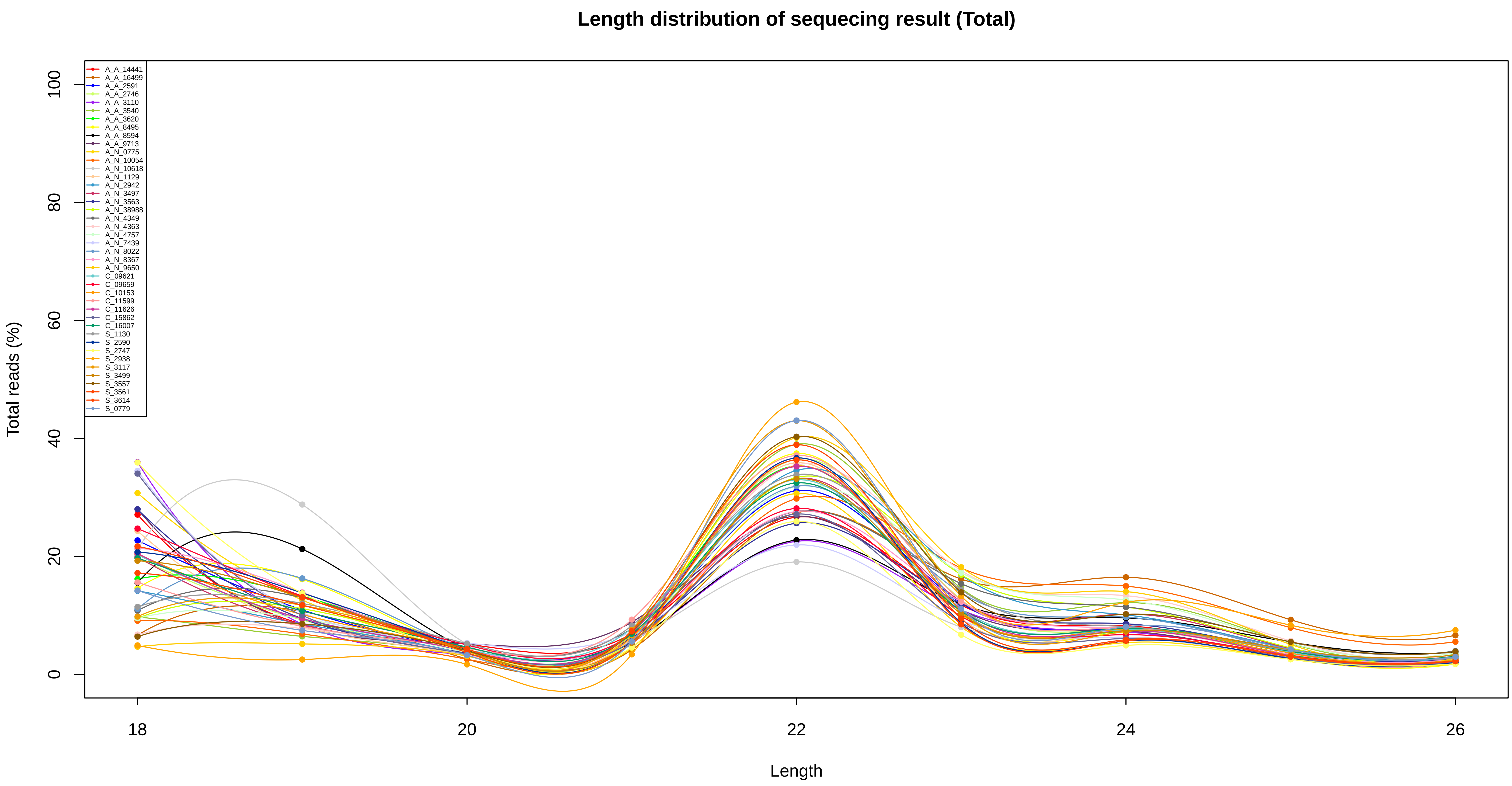
Length distribution of sequecing result (Total)
Total reads (%)
Length
0
20
40
60
80
100
18
20
22
24
26
A_A_14441
A_A_16499
A_A_2591
A_A_2746
A_A_3110
A_A_3540
A_A_3620
A_A_8495
A_A_8594
A_A_9713
A_N_0775
A_N_10054
A_N_10618
A_N_1129
A_N_2942
A_N_3497
A_N_3563
A_N_38988
A_N_4349
A_N_4363
A_N_4757
A_N_7439
A_N_8022
A_N_8367
A_N_9650
C_09621
C_09659
C_10153
C_11599
C_11626
C_15862
C_16007
S_1130
S_2590
S_2747
S_2938
S_3117
S_3499
S_3557
S_3561
S_3614
S_0779

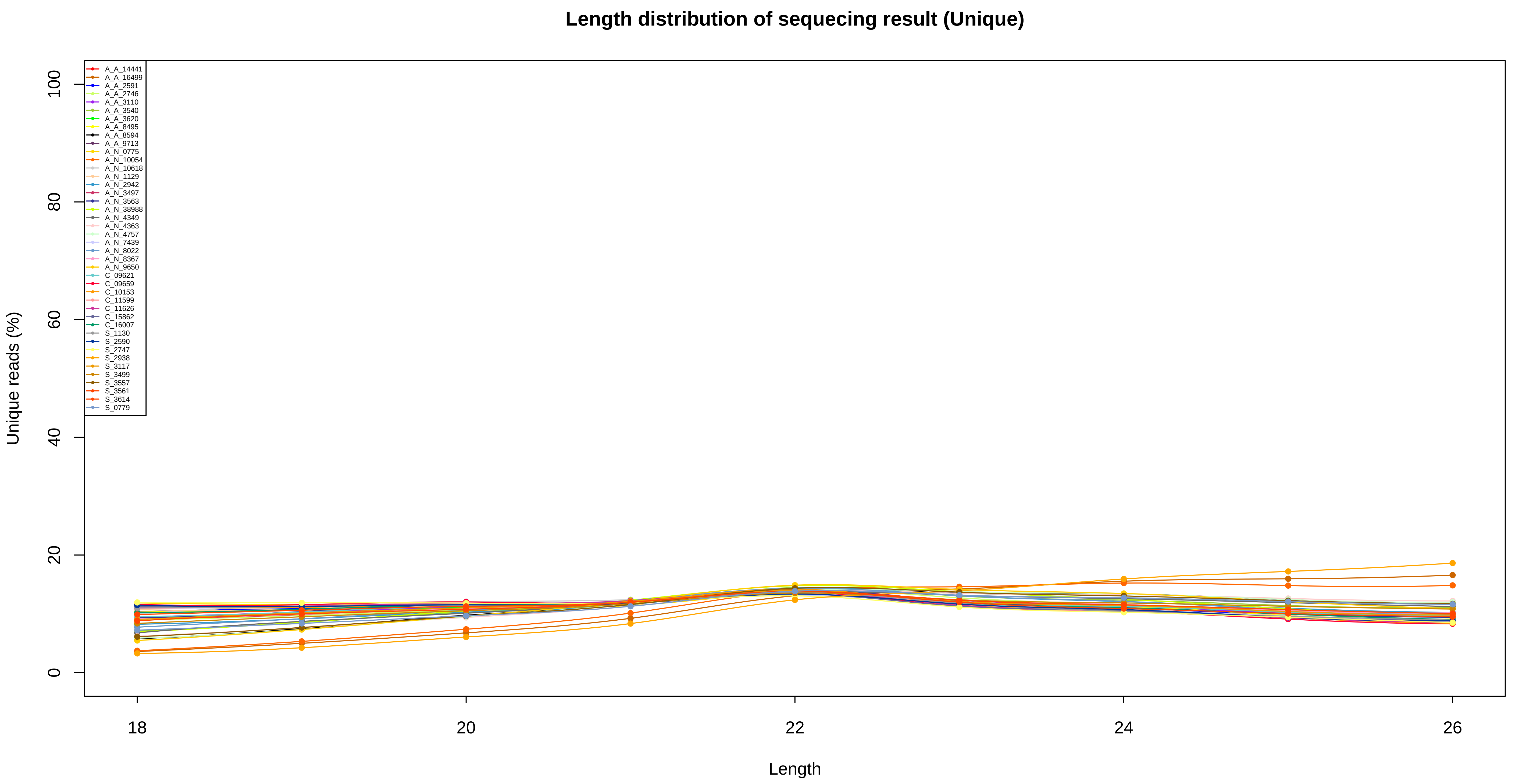

Length distribution of sequecing result (Unique)
Unique reads (%)
0
20
40
60
80
100
Length
18
20
22
24
26
A_A_14441
A_A_16499
A_A_2591
A_A_2746
A_A_3110
A_A_3540
A_A_3620
A_A_8495
A_A_8594
A_A_9713
A_N_0775
A_N_10054
A_N_10618
A_N_1129
A_N_2942
A_N_3497
A_N_3563
A_N_38988
A_N_4349
A_N_4363
A_N_4757
A_N_7439
A_N_8022
A_N_8367
A_N_9650
C_09621
C_09659
C_10153
C_11599
C_11626
C_15862
C_16007
S_1130
S_2590
S_2747
S_2938
S_3117
S_3499
S_3557
S_3561
S_3614
S_0779
