# Supplementary figures and images for: Transcriptomic Signatures of Mitochondrial Dysfunction in Autism: Integrated mRNA and microRNA Profiling
Source: Genes (Basel). 2025 Sep 10;16(9):1065. doi: 10.3390/genes16091065 (PMC12469284; doi:10.3390/genes16091065)

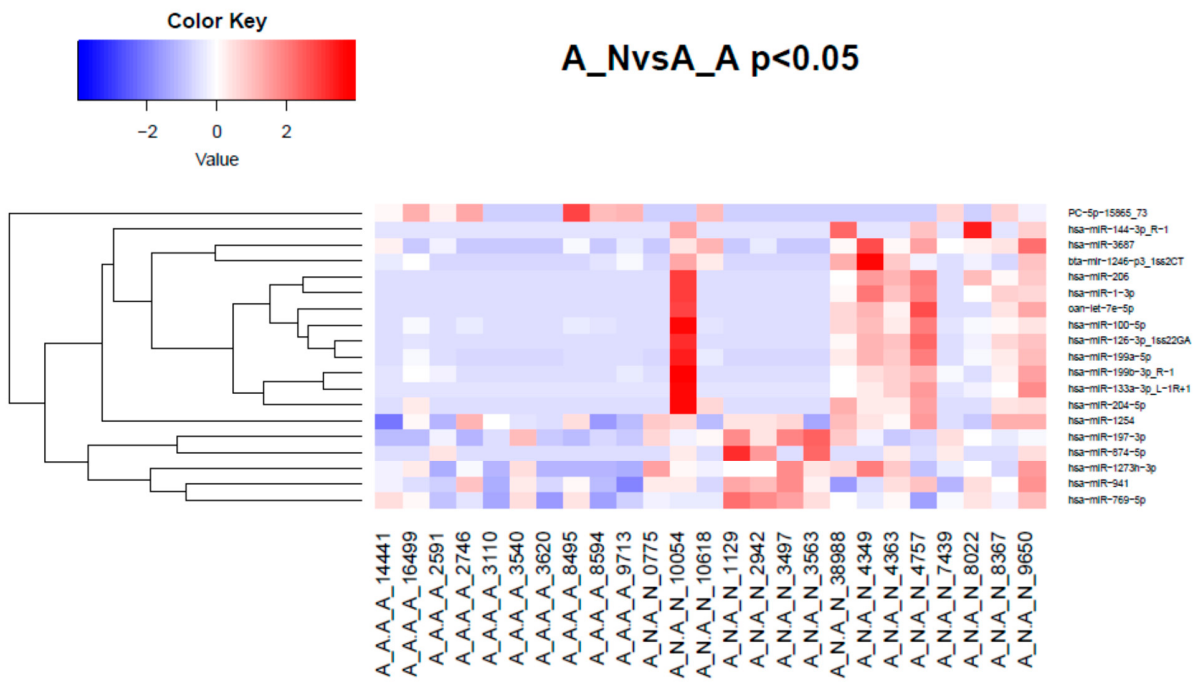

Supplement: Supplementary file 1 [file genes-16-01065-s001.zip › Supplemenary Figure S3.pdf]

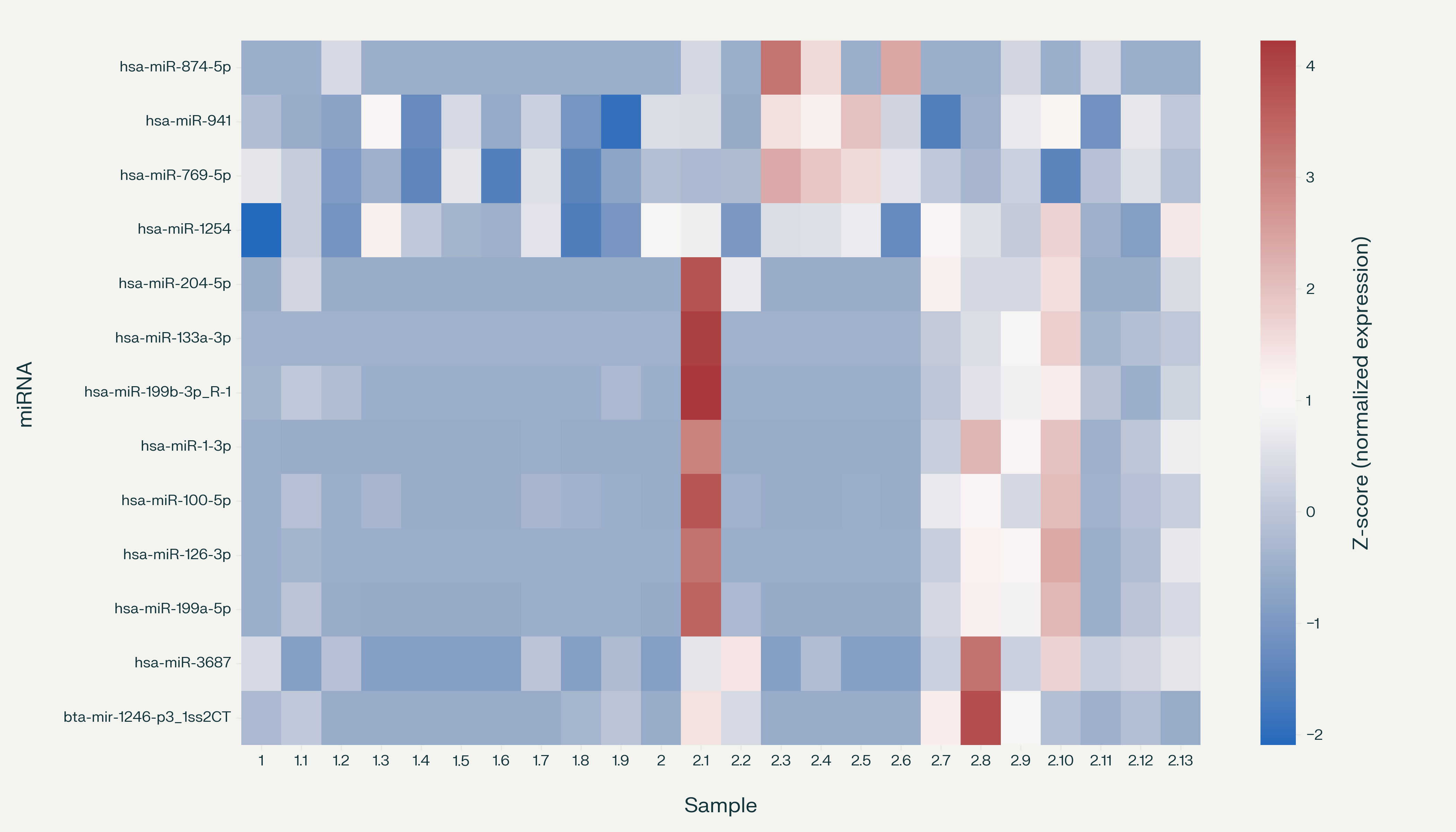

Supplement: Supplementary file 1 [file genes-16-01065-s001.zip › Supplementary Figure S4 Heatmap.png]

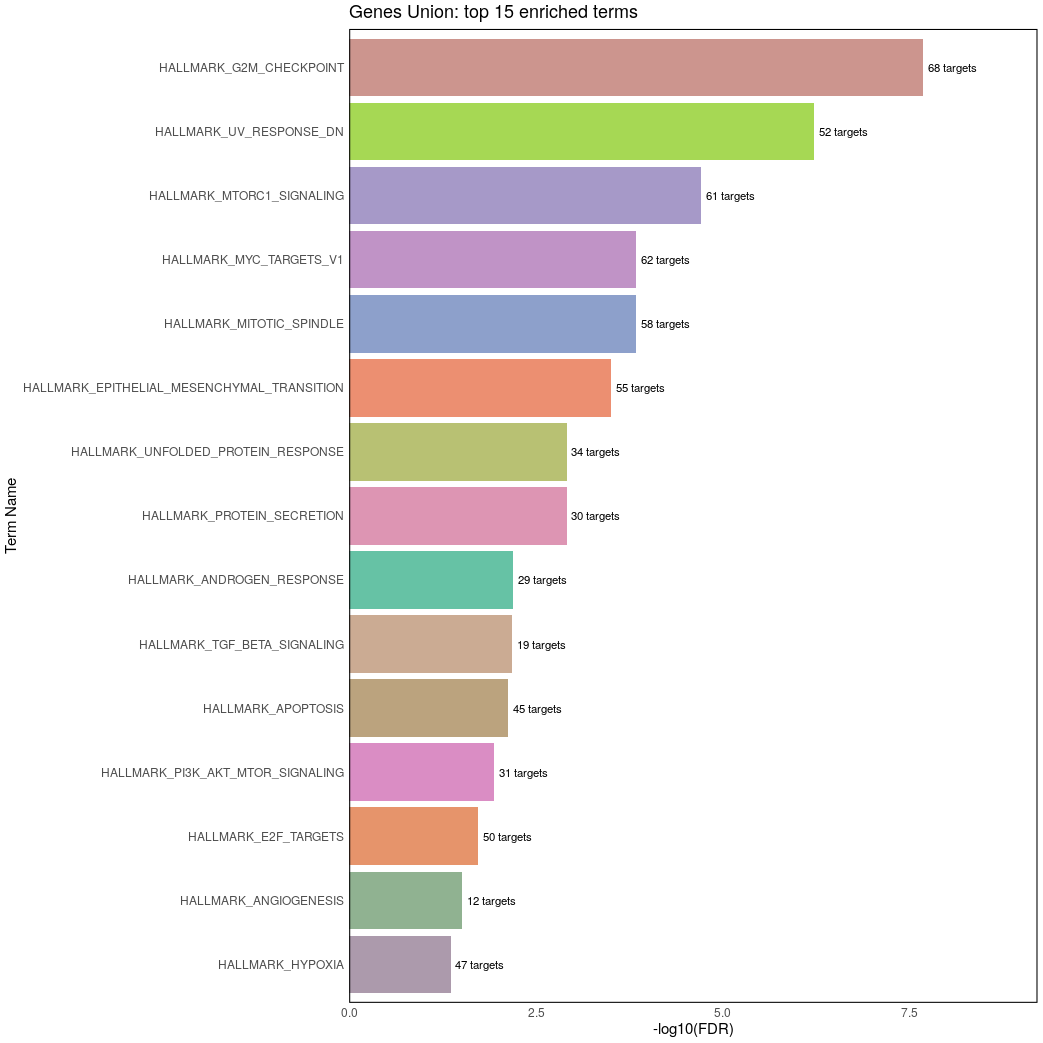

Supplement: Supplementary file 1 [file genes-16-01065-s001.zip › Supplementary Figure S5 miRNA Gene Union.png]

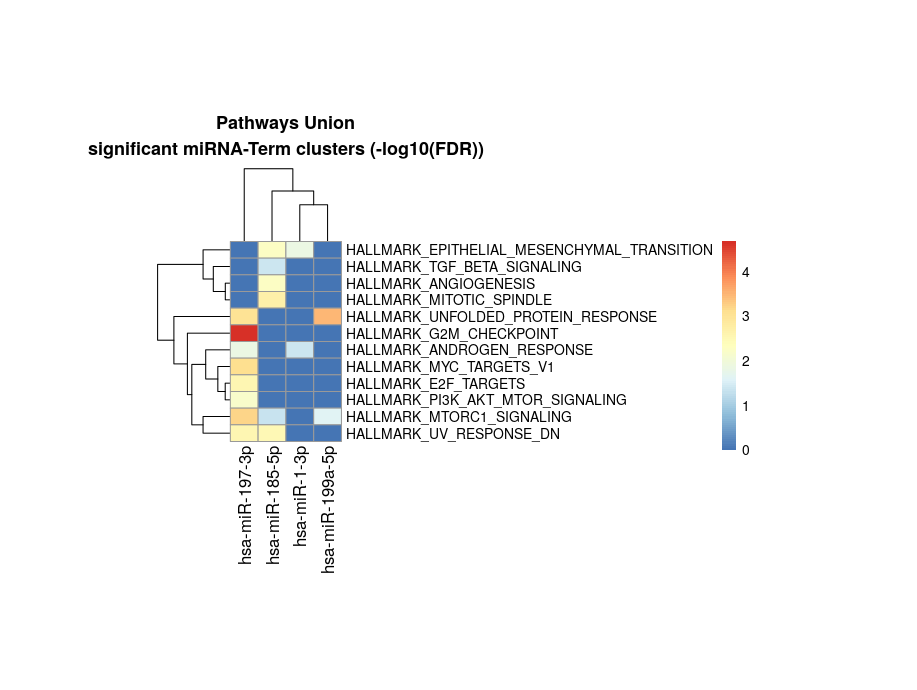

Supplement: Supplementary file 1 [file genes-16-01065-s001.zip › Supplementary Figure S6 miRNA Pathway Union.png]
